# FIBTEM provides early prediction of massive transfusion in trauma

**DOI:** 10.1186/cc10539

**Published:** 2011-11-11

**Authors:** Herbert Schöchl, Bryan Cotton, Kenji Inaba, Ulrike Nienaber, Henrik Fischer, Wolfgang Voelckel, Cristina Solomon

**Affiliations:** 1 Ludwig Boltzmann Institute for Experimental and Clinical Traumatology and AUVA Research Centre, Donaueschingenstraße 13, A-1200 Vienna, Austria; 2 Department of Anaesthesiology and Intensive Care Medicine, AUVA Trauma Centre, Dr. Franz-Rehrl-Platz 5, 5010 Salzburg, Austria; 3 Department of Surgery, University of Texas Health Science Center, 64100 Fannin Street, Houston, TX 77030, USA; 4 Division of Trauma, University of Southern California - Keck School of Medicine, 1520 San Pablo, Los Angeles, CA 90033, USA; 5 AUC Academy for Trauma Surgery, Schillerstr. 37a, 80336 Munich, Germany; 6 Division of Cardio-thoracic-vascular Surgical Anaesthesia and Intensive Care Medicine, Department of Anaesthesia, Intensive Care Medicine and Pain Medicine, Medical University, Austria Waehringer Guertel 18-20, 1090 Vienna, Austria; 7 Department of Anaesthesiology, Intensive Care and Perioperative Medicine, Salzburg University Hospital SALK, 48 Müllner Hauptstrasse, 5020 Salzburg, Austria

## Abstract

**Introduction:**

Prediction of massive transfusion (MT) among trauma patients is difficult in the early phase of trauma management. Whole-blood thromboelastometry (ROTEM^®^) tests provide immediate information about the coagulation status of acute bleeding trauma patients. We investigated their value for early prediction of MT.

**Methods:**

This retrospective study included patients admitted to the AUVA Trauma Centre, Salzburg, Austria, with an injury severity score ≥16, from whom blood samples were taken immediately upon admission to the emergency room (ER). ROTEM^® ^analyses (extrinsically-activated test with tissue factor (EXTEM), intrinsically-activated test using ellagic acid (INTEM) and fibrin-based extrinsically activated test with tissue factor and the platelet inhibitor cytochalasin D (FIBTEM) tests) were performed. We divided patients into two groups: massive transfusion (MT, those who received ≥10 units red blood cell concentrate within 24 hours of admission) and non-MT (those who received 0 to 9 units).

**Results:**

Of 323 patients included in this study (78.9% male; median age 44 years), 78 were included in the MT group and 245 in the non-MT group. The median injury severity score upon admission to the ER was significantly higher in the MT group than in the non-MT group (42 vs 27, *P *< 0.0001). EXTEM and INTEM clotting time and clot formation time were significantly prolonged and maximum clot firmness (MCF) was significantly lower in the MT group versus the non-MT group (*P *< 0.0001 for all comparisons). Of patients admitted with FIBTEM MCF 0 to 3 mm, 85% received MT. The best predictive values for MT were provided by hemoglobin and Quick value (area under receiver operating curve: 0.87 for both parameters). Similarly high predictive values were observed for FIBTEM MCF (0.84) and FIBTEM A10 (clot amplitude at 10 minutes; 0.83).

**Conclusions:**

FIBTEM A10 and FIBTEM MCF provided similar predictive values for massive transfusion in trauma patients to the most predictive laboratory parameters. Prospective studies are needed to confirm these findings.

## Introduction

Trauma-induced coagulopathy (TIC) affects 25 to 34% of all trauma patients upon emergency room (ER) admission and can be observed even before fluid resuscitation [1-3]. TIC increases the risk of massive transfusion (MT) which is associated with mortality rates up to 54% [1,4-6].

MT occurs in approximately 2 to 12% of civilian trauma patients and up to 43% of combat casualties [4,6-8]. Early identification of patients at risk of MT would minimize the risk of treatment delay. Standard coagulation tests (for example, prothrombin time (PT) or Quick value, activated partial thromboplastin time (aPTT), fibrinogen concentration) have typical turnaround times around 30 to 60 minutes or longer [9,10]. Consequently, they are of limited value for early prediction of MT; a turnaround time of ≤15 minutes would be preferable. Scoring systems for identifying patients possibly requiring MT have been developed; these include parameters such as blood pressure, heart rate and/or laboratory findings which are available without delay after ER admission [1,4,7,11-14]. However, such systems do not provide information on coagulation status.

Thromboelastometry (ROTEM^®^; Tem International GmbH, Munich, Germany) offers rapid, comprehensive assessment of the patient's coagulation status, from initiation of coagulation to the formation, quality and potential breakdown of the clot [5,15-19]. ROTEM^® ^test results have also been used to guide coagulation therapy [20-22]. We hypothesized whether ROTEM^® ^tests could potentially help identify patients who are prone to TIC and MT immediately upon arrival at the trauma centre. An investigation by Leemann *et al*. was designed to answer the same question [5], although in that study coagulation therapy was based on allogeneic blood components. In our centre we primarily use coagulation factor concentrates, which reduces transfusion of allogeneic blood products [21] and, therefore, changes the basis for predicting MT.

The primary aim of this study was to assess the predictive value of ROTEM^® ^parameters for MT among trauma patients treated with coagulation factor concentrates, with ROTEM^® ^analyses performed on samples taken immediately upon admission to the ER. The secondary aim was to compare the predictive value of ROTEM^® ^parameters with the predictive value of standard coagulation tests.

## Materials and methods

After approval from the local Ethics Committee (reference number 415-EP/73/5-2011), we performed a retrospective analysis of data from patients admitted to the AUVA Trauma Centre Salzburg, Austria, between January 2005 and December 2010. All patients with an injury severity score (ISS) ≥16, from whom blood samples were taken immediately upon admission to the ER, were eligible for inclusion in the study. Exclusion criteria were as follows: ISS <16; therapy withheld due to non-survivable injuries; patient suffered from burns; patient transferred from other hospitals. According to the decision of the local Ethics Committee, no patient informed consent was needed for this retrospective analysis.

Demographic data and clinical findings (blood pressure (BP), heart rate (HR), temperature, ISS and Glasgow coma scale (GCS) score) upon admission to the ER were collected from the anesthesia charts. ROTEM^® ^measurements (from EXTEM, INTEM, and FIBTEM tests performed on the samples taken upon ER admission) and standard coagulation test results were reviewed.

### ROTEM^® ^and standard coagulation tests

Blood samples for both ROTEM^® ^analysis and standard coagulation tests were collected in 3 mL tubes containing 0.3 mL buffered 3.2% trisodium citrate (volume ratio 1:9). Thromboelastometric analyses were typically performed at the bedside within minutes of sample collection by the attending anesthetist or intensivist. The more severe the trauma, the more quickly the analyses were undertaken (minimum set-up time approximately two minutes). Three ROTEM^® ^tests were performed: extrinsically activated assay with tissue factor (EXTEM), intrinsically activated test using kaolin (INTEM), and extrinsically activated test with tissue factor and the platelet inhibitor cytochalasin D (FIBTEM).

For the EXTEM and INTEM assays, the following variables were measured: clotting time (CT (s)); clot formation time (CFT (s)); A10 (clot amplitude 10 minutes after the end of CT); and maximum clot firmness (MCF (mm)). For the FIBTEM assay, A10 and MCF were investigated. The platelet component was calculated as MCE_EXTEM _- MCE_FIBTEM_, where maximum clot elasticity MCE = (MCF*100)/(100-MCF) [23]. Finally, the lysis index at 60 minutes (LI60 (%), clot firmness 60 minutes after CT as percentage of MCF) was collected for the EXTEM and INTEM assays. Hyperfibrinolysis was defined as complete breakdown of the clot, in accordance with previous work by our group [24].

In parallel, standard laboratory analyses were performed: Quick value and aPTT (determined on Sysmex XE-2100 (Roche Diagnostics GmbH, Mannheim, Germany)); pH, base deficit (BD) and lactate (analyzed using Roche OMNI^® ^S Blood Gas Analyzer (Roche Diagnostics GmbH); normal range for BD, −3.0 to +3.0 mmol/L; normal range for lactate 0.5 to 2.2 mmol/L). Fibrinogen concentration was measured by the Clauss method (STA-Fib^® ^assay (Roche Diagnostics GmbH); optical read-out), using a STA-Compact^® ^machine (Roche Diagnostics GmbH, Vienna, Austria). Hemoglobin, hematocrit and platelet count were analyzed using blood samples anti-coagulated with ethylenediamine tetra-acetic acid, with an SF 3000 analyzer (Sysmex Corporation, Kobe, Japan).

### Coagulation therapy, RBC administration and data management

For patients with ongoing bleeding, coagulation therapy was based on ROTEM^® ^test results with administration of coagulation factor concentrates as previously described [20,21]. Red blood cells (RBCs) were administered as required to maintain a target intra-operative hemoglobin concentration of 10 g/dL; later during intensive care unit (ICU) therapy, a lower cut-off value of 7 g/dL was accepted.

Since 2006, electronic documentation has been implemented in our hospital where the type, amount and timing of allogeneic blood products transfused are recorded. For 2005, the amount and timing of allogeneic blood transfusion were reviewed from anesthesia and ICU records. Two groups of patients were defined according to RBC transfusion: non-massive transfusion (non-MT) group (0 to 9 U RBC transfused in 24 hours), and massive transfusion (MT) group (≥10 U RBC transfused in 24 hours). This definition of MT is consistent with that used in previous publications [4,5,12,25]. There was no expectation that the two patient groups would be well matched, since patients with more severe injury upon admission are more likely to undergo MT. Also, the number of patients in the two groups was unlikely to be equal, as in our centre <50% of patients undergo MT; the inclusion of all eligible patients minimizes the risk of bias.

### Statistical analysis

Data are presented as mean ± standard deviation or median and interquartile range (IQR) for continuous variables, and as percentages for categorical variables. Continuous variables were analyzed for normal distribution by the Kolmogorov-Smirnov test. To detect differences between patient groups, either the Student's *t*-test or the Mann-Whitney U-Test was performed, depending on the underlying distribution. Group differences were compared by ANOVA with the Kruskal-Wallis test. For categorical variables, Fisher's exact test was used. Correlation between parameters was analyzed by Spearman's correlation coefficient rho. As a measure for discrimination not depending on a certain cut-off point, the area under the receiver operating characteristic (ROC) curve was determined, together with its 95% confidence interval. A *P*-value <0.05 was considered significant for all statistical tests. Statistical calculations were performed using GraphPad Prism 5.03 (GraphPad Software, La Jolla, CA, USA) and IBM SPSS 19 (SPSS Inc., Chicago, IL, USA).

## Results

During the six-year study period, 415 trauma patients were identified as eligible for inclusion in the study. Of these, 75 patients with ISS <16 and 17 patients with advanced therapy withheld due to non-survivable injuries were excluded. The remaining 323 patients were included in the study.

Patients were predominantly male (78.9%) and the median age was 44 (IQR: 26 to 59) years. Demographic data, clinical findings on admission, GCS score at site of accident and ISS for the three groups are outlined in Table 1. The MT group comprised 78 patients, while 245 patients were in the non-MT group. Patients in the MT group had significantly lower BP, higher HR, and higher ISS values upon admission to the ER compared with patients in the non-MT group. Mortality for the whole study group was 19.8% (n = 64), with a significantly higher rate in the MT group (Table 1).

**Table 1:** Demographic and clinical data on arrival at the emergency room

	non-MT group (<10 RBC units/24 hours)	MT group (≥10 RBC units/24 hours)	
	**n = 245**	**n = 78**	***P*-value**
Age (years)	43 (24 to 58)	43 (23 to 61)	ns
Male (n (%))	198 (81%)	57 (73%)	-
SBP (mmHg)	110 (90 to 130)	65 (55 to 80)	<0.0001
HR (beats per minute)	90 (77 to 110.5)	120 (100 to 125)	<0.0001
ISS	27 (20 to 34)	42 (34 to 50)	<0.0001
GCS score at accident site	12 (6 to 15)	8 (3 to 12)	0.0006
RSI in the field (n (%))	47 (19.2%)	51 (65.4%)	0.008
Temperature (°C)	35 (34 to 36)	35 (34 to 36)	ns
Mortality (n)	32 (13.1%)	32 (41.2%)	<0.0001

GCS, Glasgow Coma Scale; HR; heart rate; ISS, Injury Severity Score; MT, massive transfusion; non-MT, non-massive transfusion; RBC, red blood cell concentrate; n, number of patients; ns, not significant; RSI, rapid sequence intubation; SBP, systolic blood pressure. Data are presented as median and interquartile range or n (%). Groups were compared using the Kruskal-Wallis test.

### Differences in coagulation status between transfusion groups

#### ROTEM^® ^test results

**Figure 1:**
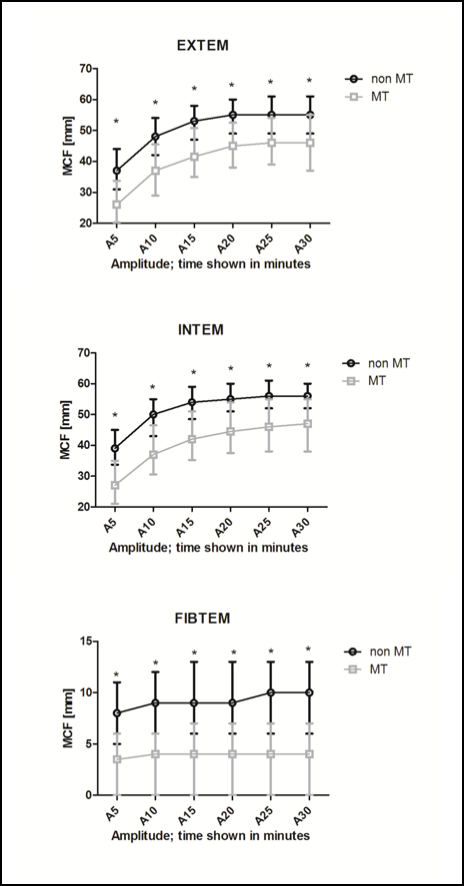
**ROTEM**^® ^**measurements differed between patients who received massive transfusion and those that did not**. Clot amplitude of EXTEM, INTEM and FIBTEM in five minute intervals for patients who received or did not receive massive transfusion. For each test, after 10 minutes, 75 to 90% of the entire MCF is reached. A5 to A30, amplitude at five-minute intervals; EXTEM, extrinsically activated thromboelastometric test; FIBTEM, extrinsically activated thromboelastometric test with cytochalasin D; INTEM, intrinsically activated thromboelastometric test; MCF, maximum clot firmness; MT, massive transfusion. Groups were compared using the Kruskal-Wallis test. * *P *< 0.0001 between groups.

ROTEM^® ^test results are outlined in Table 2. Significant differences for all ROTEM^® ^variables upon arrival at the ER were observed between the groups except for EXTEM lysis index (LI). EXTEM and INTEM CT as well as EXTEM and INTEM CFT were significantly prolonged in the MT group (*P *< 0.01). Compared with the non-MT group, patients in the MT group had significantly lower EXTEM MCF, INTEM MCF and FIBTEM MCF. The data shown in Figure 1 allow comparison of the amplitude after 10 minutes (A10) with the MCF amplitude. In the FIBTEM assay, the difference between median A10 and MCF was 1 mm in the MT group and 2 mm in the non-MT group. The platelet component was significantly lower in the MT group compared with the non-MT group (Table 2).

**Table 2:** ROTEM^® ^data on arrival at the emergency room

	non-MT group (<10 RBC units/24 hours)	MT group (≥10 RBC units/24 hours)	
	n = 245	n = 78	*P*-value
**EXTEM**			
CT (sec)	67 (56 to 90)	91 (73 to 129)	<0.0001
CFT (sec)	116 (92 to 148)	189 (128 to 264)	<0.0001
A10 (mm)	48 (42 to 54)	37 (29 to 46)	<0.0001
MCF (mm)	57 (51 to 62)	48 (41 to 55)	<0.0001
LI60 (%)	92 (88 to 95)	92 (88 to 96)	ns
**INTEM**			
CT (sec)	147 (133 to 167)	179 (148 to 220)	<0.0001
CFT (sec)	85 (68 to 109)	163 (111 to 254)	<0.0001
A10 (mm)	50 (43 to 55)	37 (31 to 47)	<0.0001
MCF (mm)	57 (53 to 62)	48 (41 to 56)	<0.0001
LI60 (%)	93 (90 to 95)	91 (73 to 94)	0.003
F**IBTEM**			
A10 (mm)	9 (6 to 12)	4 (0 to 6)	<0.0001
MCF (mm)	11 (7 to 14)	5 (0 to 7)	<0.0001
**Platelet component**			
MCE_EXTEM _- MCE_FIBTEM_	120 (95 to 148)	89 (68 to 114)	<0.0001

Data are presented as median and interquartile range. Groups were compared using the Kruskal-Wallis test. A10, clot amplitude 10 minutes after CT; CFT, clot formation time; CT, clotting time; EXTEM, extrinsically activated thromboelastometric test; FIBTEM, extrinsically activated thromboelastometric test with cytochalasin D; INTEM, intrinsically activated thromboelastometric test; LI60, lysis index 60 minutes after CT; MCE, maximum clot elasticity; MCF, maximum clot firmness; MT, massive transfusion; n, number of patients; non-MT, non-massive transfusion; ns, not significant; RBC, red blood cell concentrate.

Hyperfibrinolysis was observed in 19 patients (5.9%); 14 (75%) of these patients died. A significant difference between the study groups in LI was only observed with the INTEM test (Table 2).

#### Standard laboratory results

Values for laboratory parameters on admission to the ER are depicted in Table 3. The MT group showed severely decreased fibrinogen levels, with a median value of 95 mg/dL. Correspondingly, Quick values and aPTT showed significantly greater impairment of coagulation in the patients who received MT. Blood gas analyses and lactate levels were also significantly different between the groups (*P *< 0.0001).

**Table 3:** Standard laboratory parameters on arrival at the emergency room

	non-MT group (<10 RBC units/24 hours)	MT group (≥10 RBC units/24 hours)	
	n = 245	n = 78	*P*-value
Hemoglobin (normal range: 13.5 to 17 g/dL)	12.1 (10.7 to 13.4)	8.4 (6.9 to 10)	<0.0001
Platelet count (normal range: 150 to 350 × 10^3^/μl)	188 (158 to 234)	150 (123 to 185)	<0.0001
Quick value (normal range: 70 to 120%)	75 (65 to 88)	47 (36 to 60)	<0.0001
aPTT (normal range: 26 to 35 s)	28 (26 to 32)	45 (36 to 60)	<0.0001
Fibrinogen (normal range: 200 to 450 mg/dL)	182 (134 to 236)	95 (61.5 to 121)	<0.0001
Lactate (normal range: 0.5 to 2.2 mmol/L)	2.2 (1.3 to 3.3)	4.4 (2.6 to 6.0)	<0.0001
pH (normal range: 7.35 to 7.45)	7.33 (7.29 to 7.38)	7.23 (7.19 to 7.3)	<0.0001
Base deficit (normal range: -3.0 to 3.0 mmol/L)	3.9 (2.2 to 5.8)	7.6 (5.8 to 10.6)	<0.0001

Data are presented as median and interquartile range or as mean ± standard deviation. Groups were compared using the Kruskal-Wallis test. aPTT, activated partial thromboplastin time; MT, massive transfusion; n, number of patients; non-MT, non-massive transfusion; RBC, red blood cell concentrate.

### Prediction of massive transfusion: ROC curves

ROC curves for ROTEM^® ^parameters showed that the best predictive value for MT was provided by FIBTEM MCF (area under the curve (AUC) 0.84), with a similar outcome for FIBTEM A10 (AUC 0.83) (Table 4). For A10, a threshold of ≤4 mm provided the best values for sensitivity (63.6, 95% confidence interval (CI) 51.9 to 74.3) and specificity (82.9, CI 77.5 to 87.5). The corresponding threshold for MCF was ≤7 mm (sensitivity 78.2 (CI 67.4 to 86.8); specificity 74.7 (CI 68.7 to 80.1)). AUCs for prediction by the EXTEM and INTEM test parameters were slightly lower than those for FIBTEM MCF and FIBTEM A10 (Table 4). A statistically significant difference was observed between AUCs for FIBTEM MCF and INTEM MCF (*P *= 0.047), but there was no significant difference between the AUCs for FIBTEM A10 and INTEM MCF or EXTEM MCF. For all ROTEM^® ^parameters, the ROC-AUC remained unchanged if clot elasticity was used instead of clot firmness (for example, maximum clot elasticity (MCE) instead of MCF). The platelet component had an AUC for MT prediction of 0.74 (CI 0.69 to 0.79).

**Table 4:** Coagulation parameters and their prediction of massive transfusion (MT)

	ROC-AUC (95% CI)	Optimum threshold (for best sensitivity and specificity)	Sensitivity (95% CI)	Specificity (95% CI)
FIBTEM MCF	0.84 (0.79 to 0.88)	≤7 mm	77.5 (66.8 to 86.1)	74.9 (68.9 to 80.3)
FIBTEM A10	0.83 (0.78 to 0.87)	≤4 mm	63.3 (51.7 to 73.9)	83.2 (77.8 to 87.7)
EXTEM CT	0.71 (0.66 to 0.76)	≤72 s	76.3 (65.2 to 85.3)	59.4 (52.7 to 65.8)
EXTEM CFT	0.74 (0.68 to 0.79)	≤147 s	64.5 (52.7 to 75.1)	75.1 (69.0 to 80.6)
EXTEM MCF	0.76 (0.71 to 0.81)	≤52 mm	67.1 (55.4 to 77.5)	71.2 (64.8 to 77.0)
INTEM CT	0.71 (0.65 to 0.76)	≤167 s	65.3 (53.1 to 76.1)	75.5 (69.3 to 80.9)
INTEM CFT	0.78 (0.73 to 0.82)	≤111 s	75.0 (63.4 to 84.5)	67.3 (60.7 to 73.4)
INTEM MCF	0.78 (0.73 to 0.83)	≤51 mm	61.6 (49.5 to 72.8)	80.4 (74.5 to 85.3)
Platelet count	0.70 (0.65 to 0.75)	≤161 × 10^3^/μl	62.0 (50.4 to 72.7)	73.8 (67.8 to 79.3)
Quick value	0.87 (0.83 to 0.90)	≤60%	84.8 (75.0 to 91.9)	82.1 (76.6 to 86.8)
aPTT	0.85 (0.81 to 0.89)	≤35.2 s	71.6 (59.9 to 81.5)	87.8 (82.8 to 91.7)
Fibrinogen concentration	0.83 (0.78 to 0.87)	≤148 mg/dL	84.2 (74.0 to 91.6)	68.3 (61.8 to 74.3)
Hemoglobin	0.87 (0.83 to 0.91)	≤10.1 g/dL	77.5 (66.8 to 86.1)	84.5 (79.3 to 88.9)
Base deficit	0.76 (0.76 to 0.86)	≤6.3	69.6 (57.3 to 80.1)	79.8 (73.3 to 85.3)
pH	0.76 (0.70 to 0.81)	≤7.276	62.3 (49.8 to 73.7)	80.0 (73.6 to 85.4)
Lactate	0.74 (0.69 to 0.79)	≤4.18 mmol/L	54.9 (42.7 to 66.8)	88.0 (82.9 to 92.0)

A10, clot amplitude 10 minutes after CT; aPTT, activated partial thromboplastin time; CFT, clot formation time; CI, confidence interval; CT, clotting time; EXTEM, extrinsically activated thromboelastometric test; FIBTEM, extrinsically activated thromboelastometric test with cytochalasin D; INTEM, intrinsically activated thromboelastometric test; MCF, maximum clot firmness; ROC-AUC, area under the receiver operating characteristic curve.

Considering standard laboratory coagulation analyses, the highest predictive values for MT were provided by hemoglobin, Quick value and aPTT (ROC-AUC values shown in Table 4). Fibrinogen concentration provided predictive value similar to that of the FIBTEM parameters.

### Relationship between coagulation analyses and transfusion

Quantities for RBC transfusion and coagulation therapy within the first 24 hours are summarized in Table 5. Patients in the MT group (by definition receiving more units of RBC) also received higher amounts of fibrinogen concentrate (FC), prothrombin complex concentrate (PCC) and platelet concentrate. Fresh frozen plasma (FFP) administration is not outlined in the table as it was used in only 10 out of 323 patients.

**Table 5:** RBC transfusion and coagulation therapy administered during the first 24 hours

	non-MT group (<10 RBC units/24 hours) n = 245	MT group (>10 RBC units/24 hours) n = 78	*P*-value
RBC (U)	2 (0 to 5)	15 (12 to 17)	<0.0001
Fibrinogen concentrate (g)	0 (0 to 4)	10 (7 to 14)	<0.0001
PCC (U)	0 (0 to 0)	2,400 (1,200 to 4,275)	<0.0001
Platelet concentrate (U)	0 (0 to 0)	0 (0 to 2)	<0.0001

MT, massive transfusion; n, number of patients; non-MT, non-massive transfusion; PCC, prothrombin complex concentrate; RBC, red blood cell concentrate; U, units.

Data are presented as median and interquartile range. Groups were compared using the Mann-Whitney U-test.

**Figure 2:**
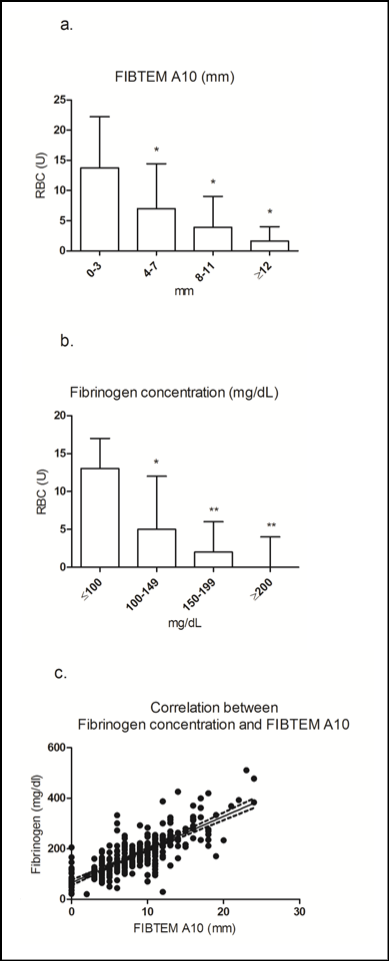
**FIBTEM A10 and fibrinogen concentration on admission to the ER, and RBC transfusion**. Low FIBTEM A10 (0 to 3 mm) and low fibrinogen concentration (<100 mg/dL) upon ER admission was associated with an increased requirement for RBC transfusion. A10, clot amplitude at 10 minutes after CT; ER, emergency room; FIBTEM, extrinsically activated thromboelastometric test with cytochalasin D; RBC, red blood cell concentrate. (a)* *P *< 0.0001 vs 0 to 3 mm; (b)* *P *< 0.001 vs ≤100 mg/dL; **P < 0.0001 vs ≤100 mg/dL; **(c) **Correlation between FIBTEM A10 and fibrinogen concentration rho = 0.75

Higher amounts of RBC transfusion were observed among patients with lower FIBTEM A10 values (*P *< 0.01) and lower fibrinogen concentration (Figure 2). Using a cut-off value for FIBTEM A10 of ≤4 mm, patients received a median of 10 (IQR 5 to 15) units of RBC, compared to a median of 2 (IQR 0 to 6) units in patients with a FIBTEM A10 >4 mm, representing five-fold higher RBC transfusion among those with the lower A10 values. Among the 31 patients with a FIBTEM A10 ≤3 mm, 26 (84%) were in the MT group. Unsurprisingly, significant correlations were observed between FIBTEM A10 and fibrinogen concentration (Spearman's correlation coefficient rho 0.78, *P *< 0.0001), and between MCF and fibrinogen concentration (Spearman's correlation coefficient rho 0.75, *P *< 0.0001). Twenty-nine patients had FIBTEM A10 values of zero and a median fibrinogen concentration of 64 mg/dL (IQR 54 to 94 mg/dL; Figure 2c). In 17 of these patients, 'spindle' traces (LI values up to 100%) for the INTEM and EXTEM assays indicated the presence of hyperfibrinolysis.

## Discussion

In this study, FIBTEM A10 and FIBTEM MCF showed similar predictive values for MT among trauma patients to those observed for the most predictive laboratory parameters (hemoglobin, Quick value, aPTT, fibrinogen concentration). Unlike the laboratory parameters, ROTEM^® ^tests provide valuable information on overall coagulation status and can be performed rapidly; consequently, they have the potential to help guide hemostatic therapy. On this basis, we advocate integration of viscoelastic testing into protocols for initial assessment of trauma patients.

Our findings are in some ways similar to those reported in a similar study by Leemann *et al*., where the same definition for MT was used and patients' demographic and clinical data were comparable [5]. Considering ROTEM^® ^parameters, elongated clot formation time and reduced clot firmness (amplitude at specific timepoints and maximum clot firmness) were observed among patients undergoing MT in both studies. The ROC-AUC for INTEM MCF in the Leemann *et al*. study (0.824) was comparable with that in the current study (0.78). However, the Leemann *et al*. study did not include FIBTEM parameters, which we report as providing better predictive value than other ROTEM^® ^parameters. Also, Leemann *et al*. reported mean RBC transfusion during the first 24 hours of 22.3 units [5]. This is higher than the median of 15 units of RBCs in our study, possibly attributable to a difference in coagulation therapy. In our study, coagulation therapy was based on fibrinogen and prothrombin complex concentrates, so that FFP was used only occasionally (10 patients out of 323). Our group has previously shown that transfusion of RBC and platelet concentrate is significantly lower when treating patients with coagulation factor concentrate-based therapy, compared with FFP-based therapy [21]. A more recent study by Davenport *et al*. was performed to identify parameters for early diagnosis of acute trauma-related coagulopathy [26]. This study reported reduced clot firmness (EXTEM assay) among trauma patients with acute coagulopathy, compared with non-coagulopathic patients. In line with findings of the present study, Davenport *et al*. also reported that EXTEM clot amplitude at five minutes may be used to predict which patients would require MT [26], although as in the study by Leemann *et al*., the FIBTEM assay was not investigated.

Most trauma patients with major bleeding are coagulopathic on admission to the ER, increasing the risk of MT [6]. Early identification of patients who require MT would enable appropriate blood products to be ordered in advance. There is typically a time delay of up to an hour between ordering FFP and having it at the bedside, ready to administer [27,28]. Thus, by ordering an appropriate quantity of FFP in advance, treatment time delays may be minimized. This could have a significant effect on patient outcomes: time to intervention appears to impact mortality to a greater extent than the FFP:RBC ratio [29].

We found that the initiation of coagulation (measured by EXTEM and INTEM CT) and the kinetics of clot formation (EXTEM and INTEM CFT) were prolonged among MT patients compared with non-MT patients. Furthermore, reduced clot quality (shown by lower MCF in all ROTEM^® ^tests) was observed in the MT group. Based on ROC curve data, FIBTEM MCF provides a higher predictive value for MT than other ROTEM^® ^parameters. Importantly, the predictive value of FIBTEM A10 was similarly high and, in addition, there were no clinically relevant differences between the median values for A10 and MCF. These findings highlight the potential for FIBTEM to provide predictive information within as little as 10 minutes after CT. This finding is novel: no previous study has assessed the potential of the FIBTEM assay to predict MT.

FIBTEM MCF or A10 is sometimes mistakenly considered as a means of measuring fibrinogen concentration. The FIBTEM assay measures elasticity of the fibrin-based clot, which is dependent not only on fibrinogen but also on other proteins such as Factor XIII [30]. Although correlations between FIBTEM MCF and fibrinogen concentration have been observed [31-33], the involvement of other proteins confounds extrapolation between these parameters. Colloid therapy (for example, hydroxyethyl starch (HES)) may be another confounder, as the presence of HES reduces FIBTEM values but increases Clauss fibrinogen concentration [34]. Additional considerations are that any FIBTEM amplitude below 2 mm is assigned a value of zero, and that fibrinolysis may potentially impair formation of the fibrin-based clot. These factors could all have contributed to our observation of zero FIBTEM A10 in 29 patients with fibrinogen concentrations up to 205 mg/dL (Figure 2c).

In the current study, the highest ROC-AUC's for MT prediction were calculated for hemoglobin, Quick value and aPTT. However, these tests are often only available after a time delay of 30 to 60 minutes or more [9,10]. Point-of-care (POC) assessment of these parameters may be undertaken, but at least for PT and aPTT this has not been investigated for predicting MT. POC measurement of fibrinogen has been attempted but this was confounded by whole-blood constituents such as RBCs and platelets [35]. Unlike ROTEM^® ^tests, laboratory parameters cannot provide timely insight into overall coagulation status or, therefore, provide valuable guidance on the best therapy to administer. In view of this and the lack of clinically important differences in predictive values for MT between FIBTEM A10/MCF and the most predictive laboratory parameters, we advocate prioritization of ROTEM^® ^analysis for trauma patients.

Time delay may explain why most scoring systems developed for activating massive transfusion protocols do not include standard coagulation tests [1,4,7,13]. For example, the trauma associated severe hemorrhage (TASH) score incorporates physiological parameters (HR and systolic BP), laboratory test results which are available within minutes (BD and hemoglobin), anatomic findings (complex long bone fractures/pelvic fracture; presence of intra-abdominal fluids) and gender. Yucel *et al*. reported an ROC-AUC for TASH of 0.88, in relation to prediction of MT [11]. Rainer *et al*. described seven variables easy to obtain in the ER, and reported an ROC-AUC for MT of 0.889 [4]. McLaughlin and coworkers used SBP <110 mm Hg, heart rate >105 bpm, pH <7.25 and hematocrit <32% to predict MT (ROC-AUC 0.74) [7]. The ABC score, reported by Nunez and Cotton, comprises four components (penetrating injury, systolic blood pressure, heart rate, positive FAST) that can be assessed within minutes (ROC-AUC 0.86) [12,13].

In contrast to these systems, ROTEM^® ^serves as a rapid coagulation monitoring system with the additional value of early MT risk stratification using the FIBTEM A10 (ROC-AUC for MT: 0.83). Taken together, the three ROTEM^® ^tests included in this study (INTEM, EXTEM, FIBTEM) enable rapid detection of most coagulation disorders [24,36]. These potentially include hyperfibrinolysis, which cannot be assessed adequately by standard coagulation tests, although it must be acknowledged that no specific definition of hyperfibrinolysis based on ROTEM^® ^parameters has yet been validated. INTEM, EXTEM and FIBTEM are routinely performed in our ER, and the ROTEM^® ^device allows them to be performed simultaneously. The results may be used to facilitate prompt, goal-directed coagulation therapy according to the patient's individual needs [20,21].

### Limitations

The clinical value of the MT definition we used in this study has been questioned: mortality increases with increasing red blood cell transfusion (no apparent threshold level of transfusion), so the criterion of 10 units in 24 hours does not allow specific selection of patients with worsened clinical outcomes [37]. An alternative definition of MT has been explored (at least five units of RBC during the first four hours) [38], but at present there is not enough evidence to support the use of such criteria.

MT patients in our study may not be directly comparable with those studied at other centres, due to our use of coagulation factor concentrates for coagulation therapy. Use of the standard FIBTEM assay also represents a possible limitation of the study: it has been shown that cytochalasin D does not provide complete inhibition of platelet activity [39]. As a result, in our study, values for FIBTEM could be higher than they should be and values for platelet component could be lower. These effects would vary between patients depending on platelet count. The retrospective nature of the study was another limitation - for example, we were not able to identify co-medications or co-morbidities that could have influenced test results. As expected, the separation of patients according to whether they underwent MT led to groups of patients that were not balanced in relation to severity of injury. This imbalance could possibly be considered as a study weakness, though on the other hand it could be considered simply as a reflection of clinical reality. The difference in patient numbers between the two groups, which is attributable simply to the number of patients meeting the inclusion criteria for each group during the study period, might also be considered as a limitation of the study. We proceeded with the analysis of the unbalanced groups because this approach avoids the bias that might potentially arise from the 'artificial' exclusion of a proportion of the non-MT patients. Similar between-group differences, both in patient numbers and severity of injury, were evident in the study published by Leemann *et al*. [5]. Lastly, the exclusion of patients for whom therapy was withheld due to non-survivable injuries could be considered as a limitation potentially introducing bias, as no patient with the maximum ISS score of 75 was included in the study. However, the inclusion of patients not receiving standard medication would certainly have introduced bias to the study, because they died without receiving any transfusion and ROTEM^® ^test results would not have been followed by any therapy.

## Conclusions

In this retrospective study of trauma patients treated with coagulation factor concentrates, FIBTEM A10 and FIBTEM MCF obtained immediately after admission to the ER provided early information on the likelihood of requiring MT. Such prediction would allow early activation of MT protocols. Through the use of a single device, viscoelastic clot testing has the potential to predict the risk of MT while also providing useful information about the underlying coagulation disorder. Taken together, these factors clearly distinguish ROTEM^® ^from other means of predicting MT such as hemoglobin level, Quick value or TASH score. Additional prospective studies are needed to confirm our data.

## Key messages

•Early identification of the need for massive transfusion (MT) may increase the speed and success of hemostatic intervention in trauma patients.

•FIBTEM MCF and FIBTEM A10 obtained from blood samples taken immediately upon admission to the ER showed similarly high predictive value for MT as the most predictive laboratory parameters.

•ROTEM^® ^analysis enables rapid detection of most coagulation disorders; this study demonstrates the additional benefit of MT risk stratification using results available within 10 minutes after CT.

•Prospective trials are required to confirm the value of ROTEM^® ^measurements in predicting massive transfusion.

## Abbreviations

A10: clot amplitude 10 minutes after CT; ANOVA: analysis of variance; aPTT: activated partial thromboplastin time; AUC: area under the curve; BD: base deficit; BP: blood pressure; CFT: clot formation time; CT: clotting time; EXTEM: extrinsically activated thromboelastometric test; ER: emergency room; FC: fibrinogen concentrate; FFP: fresh frozen plasma; FIBTEM: extrinsically activated thromboelastometric test with cytochalasin D; GCS: Glasgow Coma Scale; HES: hydroxyethyl starch; HR: heart rate; ICU: intensive care unit; INTEM: intrinsically activated thromboelastometric test; IQR: interquartile range; ISS: Injury Severity Score; LI: lysis index; MCE: maximum clot elasticity; MCF: maximum clot firmness; MT: massive transfusion; PCC: prothrombin complex concentrate; POC: point of care; PT: prothrombin time; RBC: red blood cell concentrate; ROC: receiver operating characteristic; ROTEM^®^: thromboelastometry; RSI: rapid sequence intubation; SBP: systolic blood pressure; TEG: thrombelastography; TIC: trauma induced coagulopathy; TASH-score: trauma associated severe hemorrhage score.

## Competing interests

Herbert Schöchl has received speaker's fees from Baxter, CSL Behring, Fresenius Kabi, GlaxoSmithKline and Tem International. Cristina Solomon has received speaker's fees from CSL Behring and Tem International. All other authors declare that no competing financial interests exist. Editorial assistance was provided by medical writers from Meridian HealthComms during the preparation of this manuscript. Financial support for this assistance was provided by CSL Behring.

## Authors' contributions

HS designed the study, collected the data, performed the statistical analysis and wrote the manuscript. BC and KI contributed to writing the manuscript. UN contributed to the statistical analysis and to data interpretation. HF and WV contributed to data collection and to data interpretation. CS contributed to the statistical analysis, data interpretation and to writing the manuscript. All authors have read and approved the manuscript for publication.
